# Draft Genome Sequence of Hawaiian Sea Slug Symbiont *Vibrio* sp. Strain ER1A

**DOI:** 10.1128/genomeA.00820-14

**Published:** 2014-08-21

**Authors:** Jeanette Davis, Russell T. Hill

**Affiliations:** Institute of Marine and Environmental Technology, University of Maryland Center for Environmental Science, Baltimore, Maryland, USA

## Abstract

Bacteria belonging to the genus *Vibrio* are prevalent in the marine environment and are known for forming symbiotic relationships with hosts. *Vibrio* sp. strain ER1A is a dominant symbiont of the Hawaiian sea slug, *Elysia rufescens*. Here we report the draft genome sequence of *Vibrio* sp. ER1A.

## GENOME ANNOUNCEMENT

Vibrios are a diverse group of bacteria that are widely distributed in the marine environment and form both mutualistic and parasitic relationships with a variety of hosts (1). Many of the well-studied vibrios, *V. cholerae*, *V. vulnificus*, and *V. parahaemolyticus* are responsible for human diseases, including gastroenteritis and wound sepsis (1, 2). With the exception of the mutualistic symbiont *V. fischeri* of the Hawaiian bobtail squid, there is little known about symbiotic *Vibrio* spp., that are not parasitic to their marine host. Consequently there is a need to characterized symbiotic vibrios to understand their ecological importance in relation to host function and health.

In this study we examine the genome of a symbiotic *Vibrio* sp. strain ER1A that has been continuously cultured over several years and is a major component of the bacterial community associated with Hawaiian sea slug, *Elysia rufescens* (3).

*Vibrio* sp. strain ER1A was isolated from *E. rufescens* and was grown overnight in MB2216 at 30°C with shaking. Genomic DNA from the bacterial cells was extracted using the Mo Bio Ultra-Clean Microbial DNA isolation kit (Mo Bio Laboratories Inc., Carlsbad, CA) following the manufacturer’s instructions.

*Vibrio* sp. strain ER1A was sequenced at the Institute of Marine and Environmental Technology (IMET) in Baltimore, Maryland using the Nextera XT kit with 250-bp paired-end read sequencing on an Illumina MiSeq. The 3,959,866 raw reads had an average length of 210 bps and represented 833,381,541 total bases. One hundred eight contigs (>1,000 bps) with a total size of 6,092,013 were assembled from the raw reads using CLC Main Workbench Genome Finishing Module version 7.0 (CLC Inc, Aarhus, Denmark), which resulted in about 140× coverage. The minimum contig length is 1,224 bps and the maximum contigs length is 1,593,462 bps. The overall G+C content of *Vibrio* sp. strain ER1A is 44%. The genome sequence was annotated using the NCBI Prokaryotic Genomes Annotation Pipeline (PGAP) (4) version 2.0 (http://www.ncbi.nlm.nih.gov/genome/annotation_prok/) A total of 5,202 coding sequences were predicted and 8 rRNA and 70 tRNAs were annotated. The biosynthetic gene cluster potential of *Vibrio* sp. ER1A was accessed using antiSMASH 2.0 (5) and resulted in the identification of two biosynthetic gene clusters: a putative nonribosomal peptide synthetase independent siderophore biosynthetic gene cluster and a putative homoserine lactone biosynthetic gene cluster. The presence of these types of clusters sheds light on the lifestyle within a marine invertebrate host, where both the acquisition of iron, and the coordination of community behaviors (quorum sensing) are important (6).

We have obtained a draft-genome sequence of a symbiotic *Vibrio* sp. strain ER1A, associated with the Hawaiian sea slug, *E. rufescens*. The genome sequence provides a basis for studying the potential ecological roles of non-parasitic *Vibrio* spp. associated with marine hosts, as additional genome sequences become available from these symbiotic bacteria.

### Nucleotide sequence accession numbers.

This whole-genome shotgun project has deposited in DDBJ/EMBL/GenBank under accession no. JPJA00000000. The version described in this paper is the first version, JPJA01000000.
